# Mussels drive polychlorinated biphenyl (PCB) biomagnification in a coastal food web

**DOI:** 10.1038/s41598-021-88684-9

**Published:** 2021-04-28

**Authors:** Kimberly D. Prince, Sinead M. Crotty, Alexa Cetta, Joseph J. Delfino, Todd M. Palmer, Nancy D. Denslow, Christine Angelini

**Affiliations:** 1 Department of Environmental Engineering Sciences, Engineering School for Sustainable Infrastructure and the Environment, University of Florida, 548 Weil Hall, Gainesville, FL 32611 USA; 2 Carbon Containment Lab, Yale School of the Environment, Yale University, New Haven, CT 06520 USA; 3 Department of Biology, University of Florida, Gainesville, FL 32611 USA; 4 Department of Physiological Sciences, Center for Environmental and Human Toxicology, College of Veterinary Medicine, University of Florida, Gainesville, FL 32611 USA; 5 Department of Biochemistry and Molecular Biology, University of Florida, Gainesville, FL 32610 USA

**Keywords:** Stable isotope analysis, Marine biology, Ecology, Community ecology

## Abstract

Despite international regulation, polychlorinated biphenyls (PCBs) are routinely detected at levels threatening human and environmental health. While previous research has emphasized trophic transfer as the principle pathway for PCB accumulation, our study reveals the critical role that non-trophic interactions can play in controlling PCB bioavailability and biomagnification. In a 5-month field experiment manipulating saltmarsh macro-invertebrates, we show that suspension-feeding mussels increase concentrations of total PCBs and toxic dioxin-like coplanars by 11- and 7.5-fold in sediment and 10.5- and 9-fold in cordgrass-grazing crabs relative to no-mussel controls, but do not affect PCB bioaccumulation in algae-grazing crabs. PCB homolog composition and corroborative dietary analyses demonstrate that mussels, as ecosystem engineers, amplify sediment contamination and PCB exposure for this burrowing marsh crab through non-trophic mechanisms. We conclude that these ecosystem engineering activities and other non-trophic interactions may have cascading effects on trophic biomagnification pathways, and therefore exert strong bottom-up control on PCB biomagnification up this coastal food web.

## Introduction

Decades after their ban in the United States in 1979^1,2^, high levels of polychlorinated biphenyls (PCBs), a globally pervasive persistent organic pollutant (POP), and their associated health effects in humans^3,4^ and wildlife^5,6^ continue to be reported. The lipophilic nature of PCBs enables them to bind to adipose tissues and bioaccumulate in organisms, increasing in concentration with trophic level. To date, most PCB biomagnification studies have focused on charismatic top predators, such as killer whales and dolphins^6,7^ given their susceptibility to accruing toxic-level concentrations^8^, and on commercially-valuable species that pose risks for human consumption, including salmon and tuna^9,10^. However, the sustained exposure of these guilds depends on bioaccumulation mechanisms initiated at the bottom of the food chain. Although data on lower trophic level taxa is lacking^11^, such taxa control PCB bioavailability and associated bioaccumulation rates by dictating how and where these contaminants are absorbed from the environment and incorporated into the food chain^12,13^. Therefore, despite the considerable PCB biomagnification research noted above, it is not fully understood which mechanisms, particularly those mediated by lower trophic level taxa, are primarily responsible for sustaining PCB biomagnification through food webs.

Like many other POPs, PCBs are hydrophobic and thus bind to suspended organic particles and sediments in aquatic environments^14^. Consequently, filter- and suspension-feeding taxa that ingest such particulates, including bivalves, annelids, and demersal fish, have long been used as indicators of contamination^15^. Observational surveys and lab studies have shown that these functional guilds can influence sediment PCB distribution^16–18^ and other research has shown that sediment contamination levels can affect an organisms’ bioaccumulation of PCB^19,20^. However, the degree to which filter- and suspension-feeding fauna enhance PCB bioaccumulation in other taxa through their engineering of sediment contamination has yet to be quantified or experimentally validated. For example, no work to date has assessed the effect of biodeposition of pseudofeces and feces by suspension-feeding bivalves^21,22^ on PCB bioaccumulation of infauna and epifauna that may reside within or on these biodeposits.

More generally, since biomagnification research has long-emphasized trophic transfer (i.e. food uptake) as the principle pathway by which PCBs both bioaccumulate and biomagnify across trophic levels, the importance of non-trophic interactions, such as engineering sediment contamination, in controlling PCB biomagnification across food webs is not known. Only recently have studies indicated that bioconcentration, the absorption of PCBs from water, may be a critical non-trophic bioaccumulation pathway^19,20^ and more important than biomagnification. Therefore, quantifying the influence of non-trophic interactions, such as engineering sediment contamination, in controlling PCB bioaccumulation is key to improving our ability to understand and predict subsequent biomagnification across feed webs.

**Figure 1:**
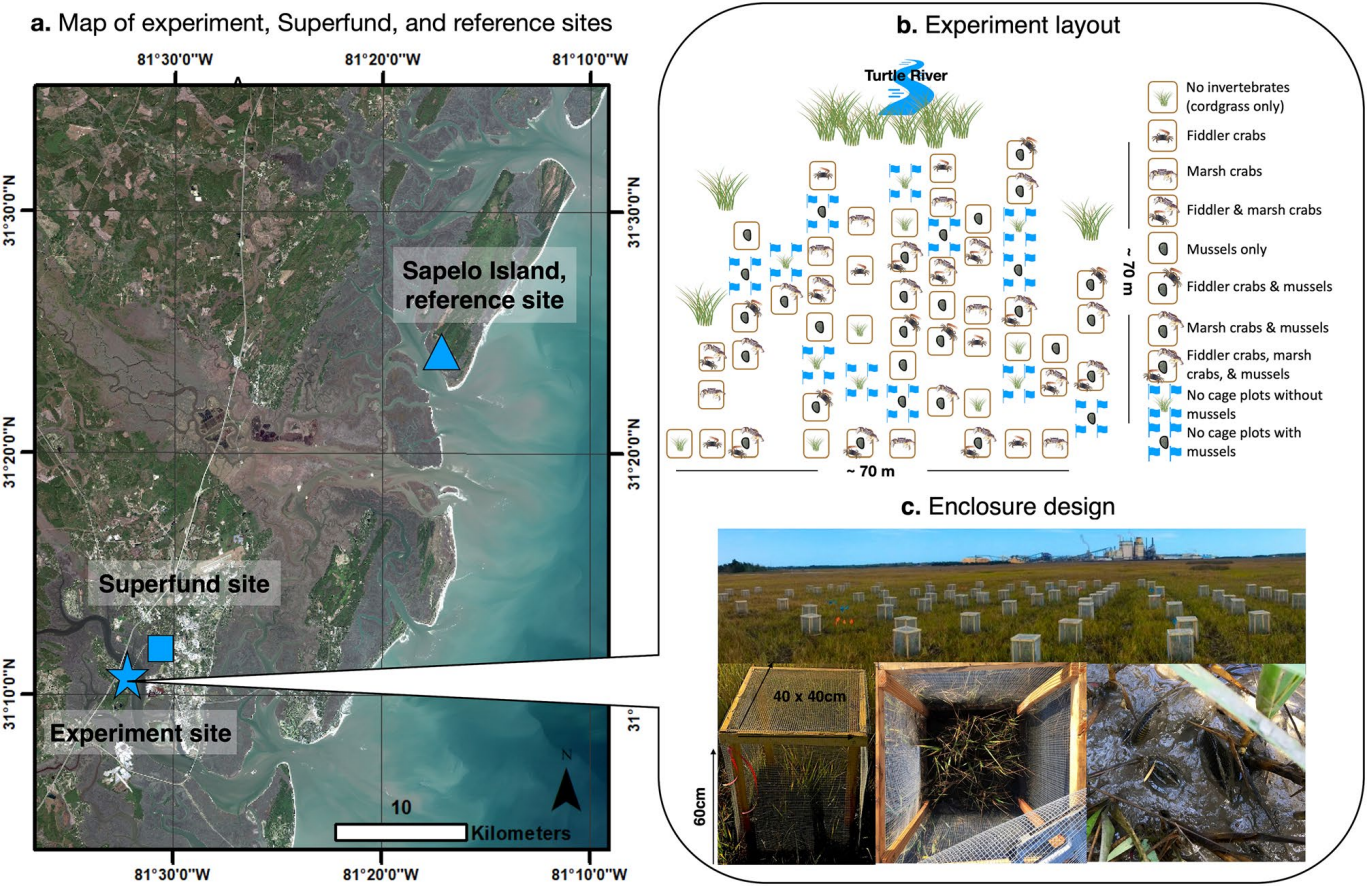
Sampling location and experimental design details. Location of experimental site on Blythe Island (blue star) in relation to the Superfund (blue square box) and reference site (blue triangle) (**a**). One of ten treatments, shown in the legend (**b**), was randomly assigned to each experimental enclosure (**c**). Icons were obtained through the integration and application network or Google Images (labeled for reuse with modification), the remaining images were photographed by K. Prince. Map was generated using ArcMap 10.7: https://desktop.arcgis.com/en/arcmap/10.7/get-started/setup/arcgis-desktop-quick-start-guide.htm.

To evaluate the role of non-trophic interactions in controlling PCB bioaccumulation, we conducted a 5-month field experiment in a southeastern US saltmarsh dominated by *Spartina alterniflora* (hereafter cordgrass). In this fully factorial experiment, we used caged enclosures to manipulate the composition of three regionally abundant macro-invertebrates: suspension-feeding, ecosystem-engineering mussels (*Geukensia demissa*, hereafter mussels), herbivorous marsh crabs (*Sesarma reticulatum*, hereafter marsh crabs), and algae-grazing fiddler crabs (*Uca pugnax*, hereafter fiddler crabs). Ecosystem engineers affect ecosystem structure and function by modifying local environmental conditions^23^. In this study, mussels serve as ecosystem engineers by modifying the physical, chemical and biological properties of sediment through their biodeposition of pseudofeces and feces. All invertebrates were collected from Sapelo Island, Georgia, an undeveloped reference site, and transported to enclosures on Blythe Island, Georgia for the duration of the experiment. We also monitored uncaged 'ambient' plots with and without transplanted mussels to evaluate possible effects of our enclosures on primary productivity and invertebrate feeding behavior. The experiment was conducted on Blythe Island in the Turtle-Brunswick River Estuary (TBRE) due to the site’s proximity (~ 2.4 km) to the EPA Superfund site located at the former Linden Chemicals and Plastics (LCP) chloro-alkali processing plant. This facility used Aroclor 1268, a highly chlorinated mixture of PCBs, from 1955 to 1994. Recent studies on fish^24^, piscivorous birds^25^, and dolphins^7^ in the TRBE, as well as nearby human populations^26^, indicate that PCB contamination is prevalent and occurs at levels potentially detrimental to some higher-trophic level species in the region (see Fig. 1 for experimental region, set-up, and design).

## Results

### Invertebrate composition effects on primary production

**Figure 2:**
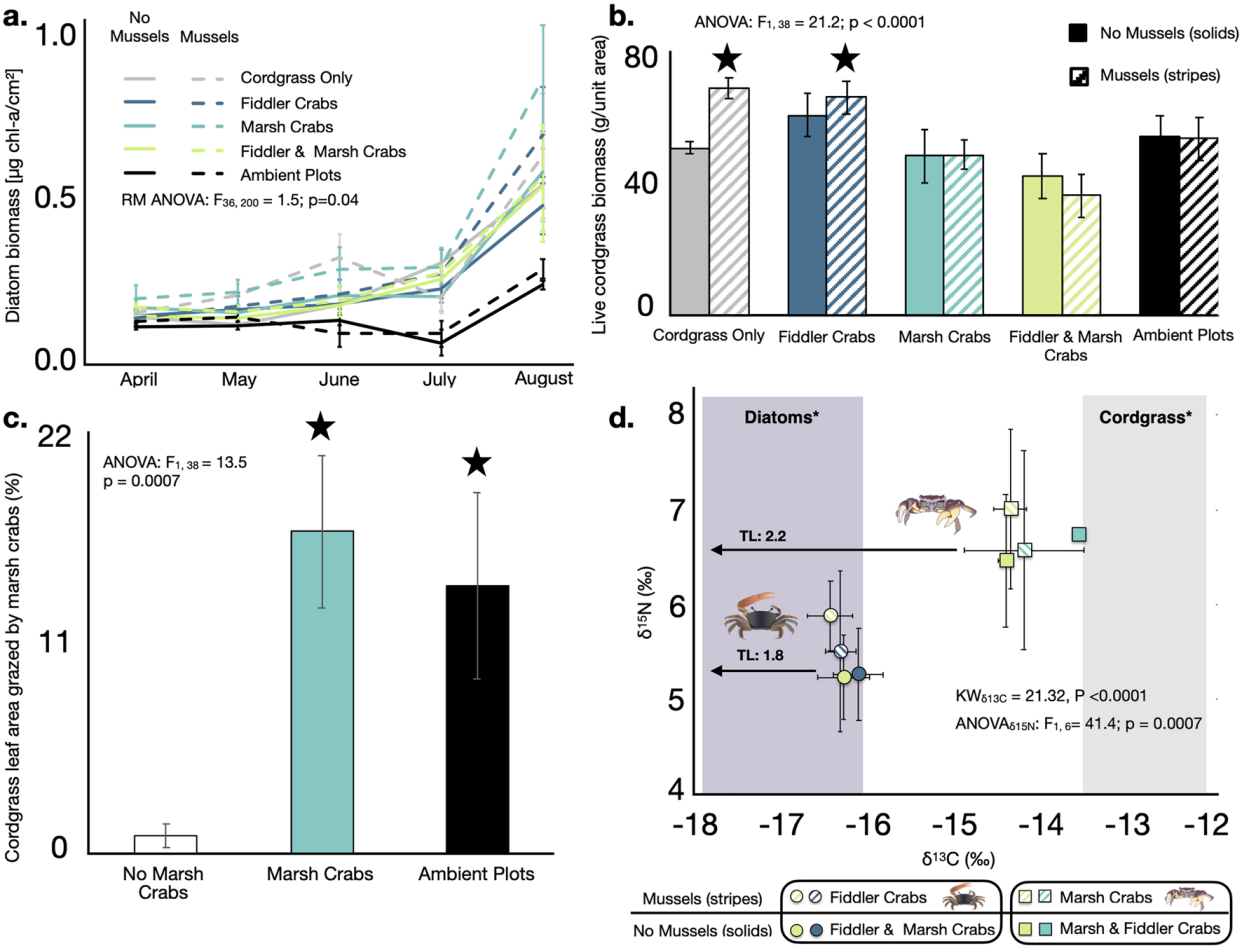
Inverttebrate composition effects on primary producer biomass, grazing damage, and crab diet. Diatom biomass recorded over the 5-month field experiment (**a**), and above ground cordgrass biomass (**b**), percent of cordgrass leaf area grazed by marsh crabs in all enclosures without marsh crabs (white), all enclosures with marsh crabs (teal) and all ambient plots (black) (**c**), and the δ^13^C and δ^15^N isotopes of fiddler and marsh crabs (**d**) recorded at the end of the experiment. Data are shown as the mean ± standard error of 3–4 measurements per plot per date in **a**; 6 replicate plots in (**b)**; 23 replicate enclosures without marsh crabs, 12 replicate enclosures with marsh crabs, and 12 replicate ambient plots exposed to background levels of marsh crabs in (**c)**; and 3 replicate enclosures per crab per treatment in (**d**). In (**d**), grey and purple shading denote the δ^13^C range previously reported for cordgrass and benthic algae, respectively, along the Georgia coast^30^. Colors and patterns denote treatment as defined in the legends for panels (**a**, **b**, **d**). Statistically significant findings (black star) are identified in (**b**, **c**). Summaries of statistical tests are shown as insets to each panel. Invertebrate icons were obtained through the Integration and Application Network or Google Imaged (labeled for reuse with modification).

To evaluate the effects of fiddler crabs, marsh crabs, and mussels on benthic algae and cordgrass production, the dietary sources for fiddler and marsh crabs, respectively^27,28^, we measured benthic diatom biomass and cordgrass stem density every 4–6 weeks and quantified cordgrass biomass and grazing damage at the conclusion of the experiment in August 2017. Diatom biomass was enhanced in enclosures with mussels and/or marsh crabs relative to enclosures with only fiddler crabs or no invertebrates, and relative to all ambient plots (F_36, 200_ = 1.5; P = 0.04; Tukey’s HSD, all P < 0.015; Fig. 2a). In addition to providing evidence of diatom consumption by fiddler crabs, these results also suggest that mussels and marsh crabs stimulate diatom production, likely through their biodeposition of nutrient-rich pseudofeces and soil aeration activities, respectively^29^.

While cordgrass stem density was not affected by treatment (P = 0.2; Supplementary Fig. S1), cordgrass biomass was 18% lower in all enclosures with marsh crabs (F_1,38_ = 21.2; P < 0.0001; Fig. 2b. Similarly, while mussels increased cordgrass biomass (Tukey’s HSD, P = 0.03), this effect was lost when marsh crabs were present (F_9, 47_ = 3.4; P = 0.003; Tukey’s HSD, P > 0.60; Fig. 2b). Grazing damage (percent of leaf area lost due to marsh crab shredding and consumption) indicated that marsh crab grazing pressure was 17-times higher in enclosures with marsh crabs and in ambient plots accessible to this species compared to enclosures lacking this species (F_1,38_ = 13.5; P = 0.0007; Tukey’s HSD, P < 0.001; Fig. 2c), reflecting both the effectiveness of our treatment design in controlling marsh crab distribution and the intensity with which this species grazes cordgrass. The low levels of grazing recorded in “no marsh crabs” plots likely occurred due to juvenile marsh crabs entering and consuming cordgrass within these enclosures given their ability to crawl through the caging mesh.

### Marsh crab and fiddler crab diet

Isotopic carbon (δ^13^C) and nitrogen (δ^15^N) analyses demonstrated that neither marsh crabs nor fiddler crabs shifted their diet (i.e. δ^13^C, Kruskal–Wallis chi-square ≥ 1.51, P ≥ 0.6) or trophic position (i.e. δ^15^N, Kruskal–Wallis chi-square ≥ 1.22, P ≥ 0.3) in response to the experimental treatments. Fiddler crab δ^13^C values (− 16.2 to − 16.5) were significantly lower than those of marsh crabs (− 13.6 to − 14.5, Kruskal–Wallis chi-square = 21.3, P < 0.0001), indicating that this species primarily consumes benthic algae [benthic algae δ^13^C: − 16.2 to − 17.9^30^]. In contrast, marsh crab δ^13^C values, although slightly lower, suggested that their diet primarily consisted of cordgrass [cordgrass δ^13^C: − 12.3 to − 13.6^30^ (Fig. 2d)]. The lower marsh crab δ^13^C values may be due to trophic discrimination factors (e.g. differences in the isotopic ratio between the consumer and its diet) which have been shown to range widely for Sesarmid species^31^. Prior work^27,28^ and our own analyses of the gut contents of > 100 fiddler and > 200 marsh crab further supported these results, indicating neither species consumes mussels or detectable quantities of other invertebrates. In addition to cordgrass, we also observed sediment particles in marsh crab stomachs. As the average δ13C for intertidal sediment sampled from a Georgia saltmarsh ranged from − 14.3%. to − 20.0%^32^, we suspect that sediments may contribute to the lower δ13C values of *Sesarma reticulatum* relative to cordgrass. Using δ^15^N values to calculate trophic level^33^, we confirm that both fiddler crabs (1.8 ± 0.04) and marsh crabs (2.2 ± 0.03) functioned as primary consumers and maintained consistent trophic pathways for PCB accumulation across treatments.

### Mussel effects on pseudofeces and sediment PCB concentrations

To assess the hypothesis that mussels enhance PCB contamination and bioavailability via their deposition of PCB-enriched pseudofeces, we collected: (1) mussel pseudofeces recently deposited on marsh surface, (2) the top 0–5 cm of root-bound sediment, (3) marsh crabs, and (4) fiddler crabs from mussel and non-mussel experimental enclosures. For each sample, we quantified the concentrations of a total of 100 PCB congeners (listed in Supplementary Table S1), hereafter referred to as PCB_T_.

**Figure 3:**
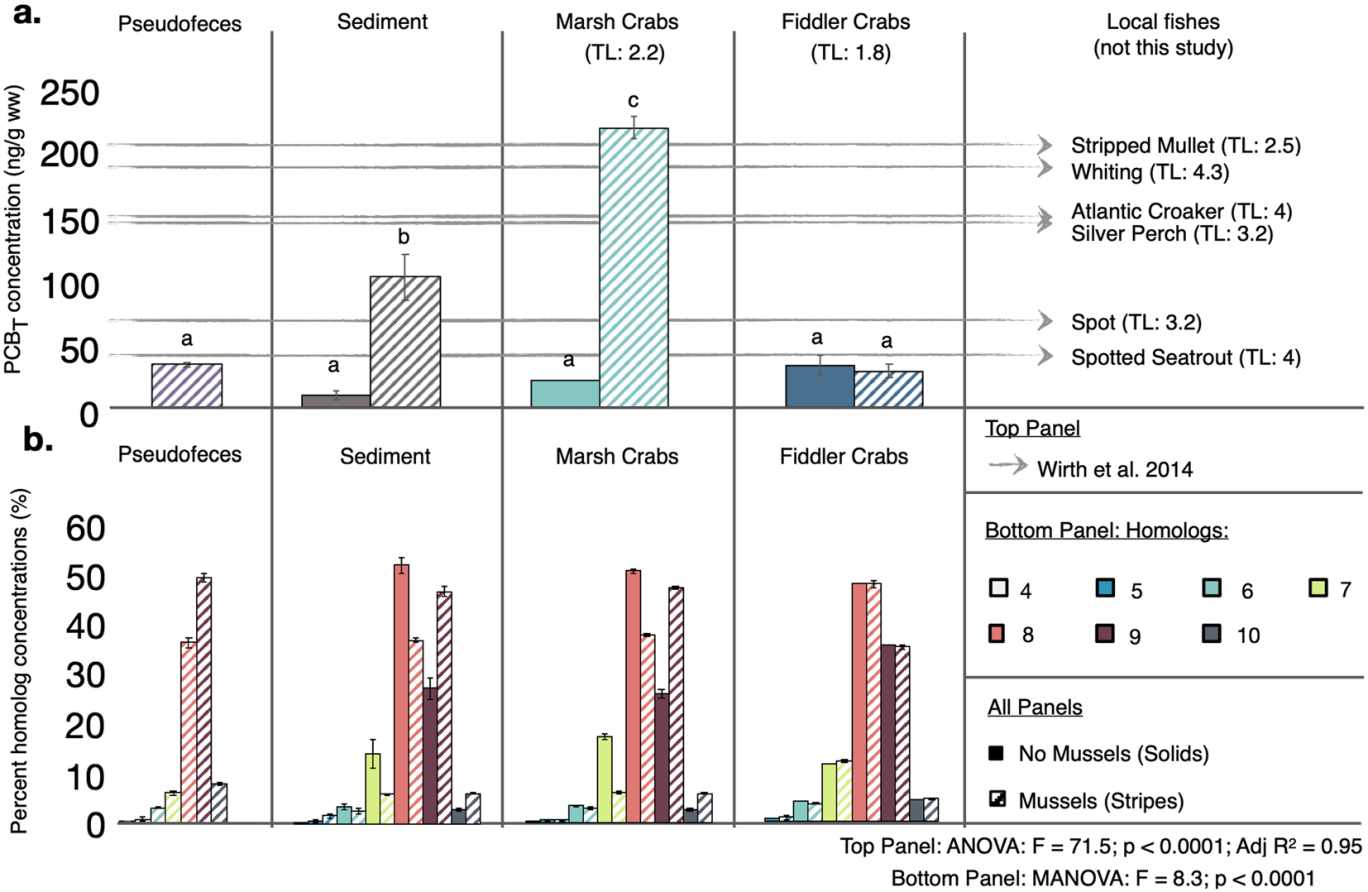
Mussel effects on PCB_T_ and PCB homolog distribution. PCB_T_, reported as the sum of 100PCB congeners in ng/g wet weight (**a**), and the percent concentration of PCB homologs Cl_4_–Cl_10_ (**b**) in pseudofece, sediment, fiddler crabs, and marsh crabs (left to right), collected from enclosures without mussels (solid bars) and with mussels striped bars. Data are shown as the mean ± standard error of 3 replicate enclosures per treatment. In (**a**), the total concentration of the same 100 PCBs measured in our study were previously recorded in stripped mullet, whiting, Atlantic croaker, silver perch, spot, and spotted seatrout filets (gray arrows; TL = Trophic Level^34^), common fish collected from the TRBE^24^.

PCB_T_ concentrations of pseudofeces [mean ± SE; 121.1 ± 6.0 ng/g dw (33.4 ± 1.7 ng/g ww)] and sediment [384.3 ± 54.4 ng/g dw (101.4 ± 17.7 ng/g ww)] in mussel enclosures were 3.7- and 11.6-times higher, respectively, than those of sediment in no-mussel enclosures [33.1 ± 8.3 ng/g dw (9.4 ± 3.0 ng/g ww); F_6,14_ = 71.5; P < 0.0001; all Tukey’s HSD P < 0.01; Fig. 3a].

The lower PCB_T_ concentrations of the pseudofeces compared to the underlying surficial sediment in mussel enclosures is likely due to intensive crab burrowing within and immediately surrounding mussel aggregations, and the effects of this burrowing on microbial activity and organic matter loss^29^. Specifically, crab burrowing aerates the soil, stimulating microbial activity and the resulting loss of organic material from the pseudofeces^29^. Since PCBs preferentially bind to organic material, we suspect that, as organic matter is lost through microbial decomposition, these contaminants gradually concentrate in the remaining, reduced volume of organic sediment particles. Over time, this material gets pushed into deeper soil depths via crab burrowing.

**Figure 4:**
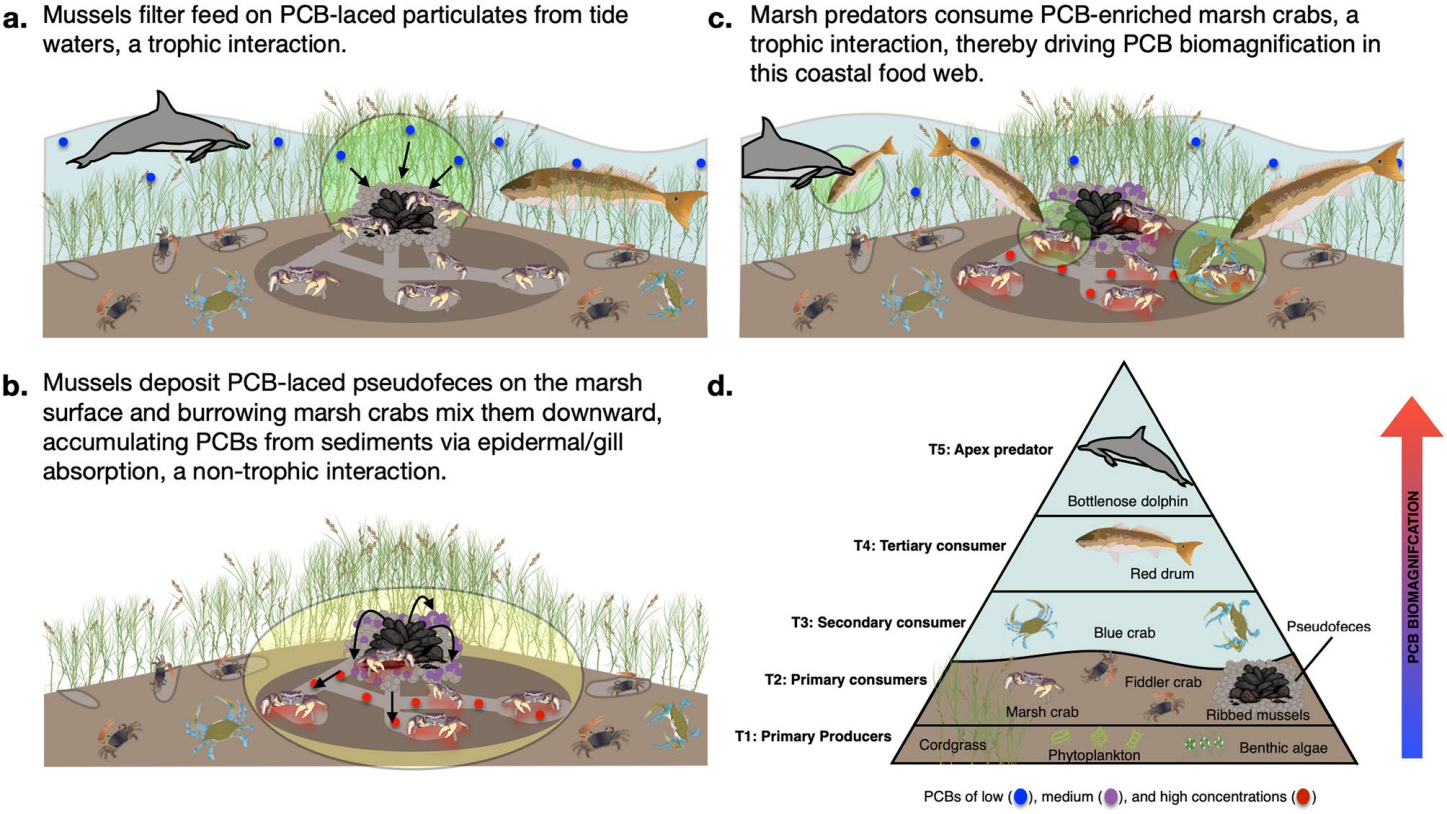
Trophic and non-trophic pathways of PCB biomagnification in saltmarsh food webs. Mussels enhance PCB concentrations in surface sediments through their filtration of PCB-laced particulates from the water column and deposition of pseudofeces (**a**). Marsh crab bioturbation, by aerating and mixing the pseudofeces into underlying marsh sediment, cause PCB concentrations to both increase and ‘sink’; and, through their sustained interaction with PCB-enriched sediment, the marsh crabs accumulate PCBs primarily through epidermal/gill absorption (**b**). Marsh crabs, as prey for nekton, then serve as vectors for PCB biomeagnification up the saltmarsh food web (**c**). Arrows signify the flow of PCBs, green and yellow circles identify trophic and non-trophic pathways, respectively, and blue, purple, and red circles denote relatively low, intermediate, and high PCB concentrations (**d**). Icons were obtainted through the Integration and Application Network or Google Imaged (labeled for reuse with modification).

The pronounced difference in sediment PCB_T_ between mussel and non-mussel enclosures supports our hypothesis that mussels rapidly elevate PCB concentrations via their filtration of PCB-enriched particulates from the tidewater and deposition of this material to the marsh surface (Fig. 4a,b).

These findings, together with surveys showing that blue mussel (*Mytilus edulis*) biodeposits increase PCB sedimentation by 50% in the Baltic Sea^18^, highlight that suspension-feeding bivalves may generate ‘hotspots’ of PCB bioavailability in coastal sediments where tidal flow velocities are low enough to enable the settling of their PCB-enriched biodeposits. By summing concentrations of 65 congeners previously reported in the region^24^ in our samples, we also found that sediment PCB concentrations in mussel enclosures (238 ± 35.7 ng/g dw) were nearly 3-times greater [and in non-mussel enclosures 5-times less (16.3 ng/g dw)] than the average total PCB concentration reported for the top 2-3 cm of archived saltmarsh sediment collected near the LCP facility in 1996, the year it was placed on the National Priorities List (79.3 ± 2.47 ng/g dw)^24^. Thus, mussels enhanced PCB concentrations in our enclosures to “superfund site-levels” within 5 months, highlighting the magnitude of their localized effects on enhancing PCB bioavailability.

### Mussel effects on crab PCB biomagnification

After 5-months on Blythe Island, fiddler crabs accumulated PCB_T_ concentrations approximately 30-times greater (33.0 ± 6.8 and 28.4 ± 5.1 ng/g ww in no-mussel and mussel enclosures, respectively) than those collected directly from our reference site, Sapelo Island (PCB_T_: 1.1 ± 0.02 ng/g ww, Fig. 3a, Supplementary Fig. S2). However, fiddler crab PCB_T_ did not differ across treatments (P > 0.9, Fig. 3a). Thus, fiddler crabs responded to the enhanced PCB exposure on Blythe Island relative to our reference site but were insensitive to the localized mussel-induced enhancement of PCBs in pseudofeces and sediment.

In contrast, marsh crabs from mussel enclosures (217.9 ± 8.8 ng/g ww) were significantly greater with concentrations on average 43-times higher-than their baseline values from Sapelo Island (5.1 ± 4.3 ng/g ww) and over 10-times higher than marsh crabs from no-mussel enclosures (20.8 ± 7.4 ng/g ww, Tukey’s HSD, all P < 0.001, Fig. 3a, Supplementary Fig. S2). Further, marsh crab PCB_T_ from mussel enclosures was comparable to, or far exceeded, the average values reported for several higher-trophic level fish in the TBRE^24,34^ (Fig. 3a). As marsh crabs are important prey for many of these fish and other predators (e.g. birds^35^, red drum^36^), our data suggest that this primary consumer, when associated with mussel mounds, has the potential to be an important vector sustaining PCB biomagnification in this coastal food web (Fig. 4c,d).

### PCB homolog composition analyses

To confirm the principle role of mussels in driving increased sediment and marsh crab PCB_T_ concentrations via their pseudofeces, we compared homolog (Cl) profiles. Homologs are classes of PCBs based on their number of chlorine atoms, which range from one to ten. Homolog one (Cl_1_) consists of PCBs with one chlorine atom whereas Cl_10_ consists of PCB 209, the only congener with 10 chlorine atoms. Highly chlorinated congeners are more persistent and more prone to biomagnification^37^. While all samples were dominated by the highly chlorinated homologs (Cl_7_–Cl_10_) associated with Aroclor 1268^7,24,38^, the pseudofeces exhibited a distinct profile, which was highly dominated by Cl_9_ and had the highest proportion of Cl_10_, relative to other sampled media. Within mussel enclosures, sediment and marsh crabs exhibited identical profiles (Tukey’s HSD, P > 0.40), being similarly dominated by Cl_9_ and high proportions of Cl_10_. Sediment and marsh crabs from no-mussel enclosures and all fiddler crabs were also indistinguishable (Tukey’s HSD, P > 0.30), and were instead dominated by Cl_8_ and exhibited relatively low proportions of Cl_10_ (MANOVA; F_5,12_ = 7.3, P < 0.0001; Fig. 3b).

Collectively, these homolog profiles suggest that the particulate-bound PCBs in tidewater that mussels filter and deposit as pseudofeces are more chlorinated than the PCBs present in marsh surficial sediments, which likely express lower chlorination due to environmental degradation processes^37^. Through the biodeposition of these highly chlorinated PCBs, mussels not only increase PCB_T_, but also redistribute highly chlorinated PCBs to surficial sediments. Moreover, the identical homolog profiles of marsh crabs and sediments in both mussel and no-mussel enclosures indicate that this species primarily accumulates PCBs via epidermal or gill absorption from contaminated sediment, a non-trophic pathway, rather than through trophic transfer (i.e. cordgrass consumption) (Fig. 4c). If marsh crabs were accumulating PCBs solely from cordgrass consumption, one would expect their homolog profile to be less chlorinated than the sediment profile because cordgrass selectively accumulates less chlorinated PCB congeners relative to the sediment in which it grows^39^. Thus, marsh crab bioaccumulation of highly chlorinated PCBs most likely occurs through the non-trophic mechanism of absorption from the sediment rather than through trophic transfer.

Our finding that fiddler crabs exhibit similar homolog profiles regardless of mussel presence suggest that this species, unlike marsh crabs, did not interact strongly with the pseudofeces or sediment adjacent to mussel aggregations (i.e. where sediment was sampled). Indeed, prior research has shown that fiddler crabs of the size transplanted into our experiment rarely burrow immediately on or near mussel aggregations^22^. The fiddler crabs’ lack of attraction to mussel aggregations and the marsh crabs’ strong affinity for burrowing adjacent to mussels^29^ highlights that niche differences among functionally similar organisms can dramatically influence which species are most prone to biomagnification via this non-trophic pathway.

### Impact of mussels on risk of adverse biological effects

Finally, to determine whether the PCB_T_ recorded in mussel versus non-mussel enclosures are high enough to induce adverse biological effects, we first compared our sediment concentrations with effects range low (ERL) and median (ERM) values. Concentrations equal to or below the ERL (22.7 ng/g dw) rarely result in adverse biological effects whereas those equal to or above the ERM (180 ng/g dw) are likely to induce deleterious biological effects^40^. While 100% of pseudofeces and sediment from mussel enclosures surpass the ERM value, only 30% of the sediment sampled in no-mussel enclosures exceeded this threshold, suggesting that mussels increased the risk of adverse biological effects among the benthic and surrounding estuarine community in only a few months’ time.

### Impact of mussels on local dioxin-like toxicity

To further explore the potential adverse health impacts of PCB concentrations on marsh and fiddler crabs, we summed the concentration of the 12 most harmful PCBs whose toxicity is similar to polychlorinated dibenzo-p-dioxins^41^. Of these Dioxin-Like PCBs only five (105, 118, 126, 169, 189, hereafter referred to as PCB_DL_) were detected above our analyte-specific method detection limit and thus included in our PCB_DL_ values. Marsh crab PCB_DL_ from mussel enclosures (4.7 ± 0.24 ng/g ww) was significantly greater compared to all other treatments and mediums sampled (F_6,14_ = 18.7, P < 0.0001), which were indistinguishable from each other with PCB_DL_ ranging from 0.03 (Sapelo Island marsh crabs) to 1.8 ng/g ww (sediment from mussel enclosures) (Supplementary Table S2).

Dioxin-like toxicity is commonly expressed by a toxic equivalency value (TEQ), which is the sum of the concentration of each dioxin-like compound detected in the sample multiplied by its corresponding toxic equivalent factor (TEF)^41^. TEFs are a ratio of the half maximal effective dose (ED50) for Tetrachlorodibenzo-p-dioxin (TCDD), a highly toxic dioxin, to the ED50 for the dioxin-like compound of interest^41,42^. Using standard TEFs^41^, we found that TEQs were 4- and 13-times greater in sediment and marsh crabs from mussel relative to no-mussel enclosures (Supplementary Table S2). Further, marsh crab TEQs increased 18-times in no-mussel and 157-times in mussel enclosures on Blythe Island compared to their baseline TEQs from Sapelo Island (Supplementary Table S2) and were significantly higher than all other sampled media (F_6,14_ = 23.26, P < 0.0001). Fiddler crab TEQs also increased 30- and 20-fold in no-mussel and mussel-enclosures relative to their Sapelo Island baseline values (Supplementary Table S1).

Since no TEQ data was found in the literature for either crab species in this study, we compiled PCB TEQs from five similar invertebrates in the literature (Supplementary Table S2) to contextualize our results. These PCB TEQs ranged from 0.2 pg/g ww (blue crab, *Callinectes sapidus* from Pensacola, FL^43^) to 39.6 pg/g ww (Chinese mitten crab, *Eriocheir sinensis* from Thames River, UK, where historically there have been high PCB inputs^44,45^). The average marsh crab TEQs from mussel enclosures surpassed those reported for the Chinese mitten crab, a burrowing omnivore, by threefold (131.9 pg/g ww) while the rest of our study’s TEQs fell within the range reported in the literature.

Collectively, our ERL, ERM, and TEQ values, all standardized metrics of risk for PCB-associated adverse health effects, highlight that mussels, through their filtering and deposition of highly chlorinated PCBs from tidewaters to the marsh surface, are dictating where PCB concentrations are continuing to reach biologically-harmful levels on Blythe Island and, likely, in saltmarshes throughout the TRBE and surrounding region more generally.

## Discussion

This study is the first to experimentally expose the role of mussels, through their engineering of localized surficial sediment contamination, in rapidly (i.e. 5-months) augmenting PCB bioaccumulation in fiddler and marsh crabs, thereby fueling PCB integration into coastal food webs (Figs. 2, 4). Due to niche partitioning, fiddler crabs, which do not commonly spatially overlap with mussel aggregations^22^, exhibited little response to mussel-mediated enrichment of sediment PCBs while marsh crabs, which often burrow adjacent to mussel aggregations, were highly responsive to the elevated PCB concentrations in mussel-engineered sediments. In fact, when in mussel enclosures, marsh crabs accumulated PCBs at levels similar to or even greater than those reported for higher trophic level fish in this coastal food web^29^ (Fig. 3A). In conjunction with our homolog analyses, these data suggest that marsh crabs primarily bioaccumulate higher chlorinated PCBs via absorption through their epidermis or gills, from the sediment rather than through cordgrass consumption. Altogether, this study establishes a new frontier in contaminant fate and biomagnification research focused on the dual role of trophic and non-trophic interactions in mediating bioaccumulation pathways of PCBs and many other persistent organic pollutants.

Our field experiment also revealed the potential for mussels to influence both the location and magnitude of the detrimental health effects of PCBs in southeastern US estuaries. Mussels drove sediment PCB concentrations above the ERM—i.e., levels likely to induce adverse biological effects—in 100% of samples at the conclusion of our 5-month experiment. In contrast, 70% of sediment samples from areas without mussels were below the ERL, i.e., levels that rarely result in adverse biological effects. Further, marsh crabs in mussel plots exhibited TEQ values an order of magnitude greater than other invertebrate TEQ values found in the literature to date (Supplementary Table S2), which is likely due the marsh crabs’ strong preference for burrowing near mussel mounds. Further research is required to evaluate the effects of these mussel-induced PCB concentrations on the fitness of marsh crabs and other fauna in this system. This relationship may be leveraged by future studies to identify where to focus such research efforts.

In this study, we focused on mussels because of their numerical abundance (mussel aggregations cover up to 15% of the marsh surface^29^ ), geographically widespread distribution (i.e. this species of mussel occurs from Florida to Maritime Canada), and high degree of predictability regarding their spatial distribution within and across saltmarsh habitats^46^. The size and spatial coverage of mussel aggregations increases with the length of adjacent tidal creeks, which may be utilized by future studies to predict where mussels have the strongest, landscape-scale impacts on sediment and benthic invertebrate PCB levels, as well as the trophic transfer of PCBs up coastal food webs to higher trophic level predators. Such higher trophic level species that depend on saltmarsh invertebrate prey include red drum (*Sciaenops ocellatus*), sheepshead (*Archosargus probatocephalus*) and blue crabs (*Callinectes sapidus*), which comprise both culturally and economically important recreational and commercial fisheries in this region.

Given that suspension-feeding and sediment-sifting taxa are numerically abundant components of almost all freshwater and marine ecosystems (e.g. bivalves, sponges, worms, corals, and some fishes), the non-trophic mechanisms exposed in this study are likely transferrable and important modulators of persistent organic pollutant fate and transport in many other aquatic systems. Based on our findings, such organisms likely exert a similar, non-trophic influence on contaminant concentrations. We hypothesize that sessile organisms in low flow environments will have the greatest effect on local contamination regimes, as their biodeposits have the potential to settle and accumulate in place. This study therefore serves as launching point for more research dedicated to evaluating the relative effects of these different species in controlling the bioaccumulation and biomagnification of PCBs and other similarly behaved persistent organic pollutants. Advancing knowledge of the relative efficacy and importance of these different suspension-feeders in controlling biomagnification pathways in various environmental contexts is an exciting frontier for developing solutions to the continued global transport and biomagnification of both currently manufactured and long regulated POPs.

## Methods

### Experimental set-up

On April 2017, 40 cm × 40 cm × 60 cm (length × width × height) experimental enclosures were spaced 2–4 m apart in a 70 m × 70 m area of saltmarsh on Blythe Island where we randomly assigned each plot one of ten treatments: (1) caged cordgrass only (e.g. no invertebrates), (2) caged fiddler crabs, (3) caged marsh crabs, (4) caged fiddler crabs + marsh crabs, (5–8) all above caged combinations + mussels, (9) uncaged ambient control with no mussels, 10) uncaged ambient control with mussels (n = 6 replicates per treatment). Enclosure cages consisted of four wooden stakes, which were secured in the ground for stability and 6.35 mm mesh hardware cloth which covered all four sides and the top. Prior to cage installation, we removed a 40 × 40 × 20 cm (length by width by depth) ‘block’ of marsh soil and vegetation located within each cage, and placed fiberglass window screen mesh (1.5 mm mesh size) along the bottom and sides of each block. This cage design maintained the composition of macro-invertebrates transplanted into each enclosure by preventing crabs (all > 10 mm at the start of the experiment) from exiting or entering while allowing free exchange of tidal flow and porewater. Prior to the start of the experiment, all resident macro-invertebrates were gently removed from each enclosure by hand to ensure the only macro-invertebrates occurring within the cage were those we transplanted.

All transplanted experimental organisms were collected from saltmarshes surrounding Sapelo Island (31° 23′ 56.1″ N, 81° 17′ 18.5″ W; Fig. 1b), a largely undeveloped Georgia barrier island located 34 km north of Blythe Island (see Supplement for additional site details). We stocked experimental enclosures with invertebrates at densities that were based on those commonly observed in healthy saltmarshes in the area^47^: n = 6 marsh crabs, n = 20 fiddler crabs, and n = 15 mussels per enclosure^29^. In mussel enclosures, we transplanted 5 small (30–49 mm), 5 medium (50–69 mm) and 5 large mussels (70–80 mm) in a clustered design to mimic their natural distribution in aggregations^22,29,48^. We collected 623 fiddler crabs and 287 marsh crabs from Sapelo to both stock all of our enclosures and to provide material for baseline analyses of PCB concentrations prior to their introduction to our experimental site.

### Sample collection

Every 4–6 weeks over the duration of the experiment, we counted live and dead cordgrass stems and measured the height of 10 randomly selected stems. During this time, we also recorded three to four measurements of benthic algae biomass (e.g. diatoms) in all plots using a handheld fluorometer (BenthoTorch, bbe Moldaenke GmbH, Schwentinental, Germany). We also monitored invertebrate densities by counting the number of live and dead mussels, fiddler crab burrows, and marsh crab burrows^27^. We counted burrows instead of individual crabs because it would have been highly destructive to probe crabs out of their burrows and because burrows provide fairly accurate assessments of crab density^27^. The average number of fiddler crab and marsh crab burrows did not significantly differ by date and mussel mortality was insignificant among enclosures throughout the duration of the experiment, therefore suggesting invertebrate densities remained constant. To assess whether temperature and salinity levels differed between caged and ambient plots, we measured soil temperature with a thermometer and porewater salinity using a handheld refractometer^47^ via rhizon porewater samplers that were inserted in the marsh surface (0–5 cm) during low tide when the marsh surface was not inundated. The number of live and dead stems, stem height, salinity, and soil temperature did not differ among plots (Tukey’s HSD, all P > 0.05).

After 5 months, a handheld metal corer was used to collect 2 cm diameter cores of the top 0–5 cm of sediment (n = 3 replicate cores from each enclosure). In mussel-addition enclosures, cores of recently deposited pseudofeces located immediately adjacent to the mussels were also collected (n = 3 replicate cores of pseudofeces from each mussel enclosure). The corer was thoroughly rinsed with DI water between samples. All mussels, marsh crabs and fiddler crabs were then retrieved from enclosures as well as all mussels from ambient plots. Invertebrates, sediment, and pseudofeces were stored in solvent-rinsed mason jars and put on ice during the transport back to the University of Florida, Gainesville, FL. In the laboratory, invertebrates were rinsed with DI water and all samples were stored at − 20 °C. All cordgrass stems were also harvested from each plot, separated into live and dead stems, rinsed, counted, and measured for length. Leaves were then visually scanned for marsh crab grazing wounds (which are distinct given the shredded margins they leave behind) after which the percent of leaf area consumed by marsh crabs was estimated and recorded. Live cordgrass stems were then pooled for each plot, dried, and weighed for biomass.

### Stable isotope analysis

For stable isotope analyses, with the exception of enclosures with only marsh crabs, we used crabs from three of the six enclosures per treatment while samples from the remaining three plots were reserved for PCB analyses. Due to limited recovery of marsh crabs from marsh crab-only treatments, marsh crabs from only one plot of this treatment were used for stable isotope analyses whereas marsh crabs from three plots of this treatment were used for PCB analyses. We removed the carapace and gills for all crab samples per enclosure, rinsed their tissues with DI water, and collected all soft muscle tissues from their body and legs. Samples were dried in the oven at 60 °C for 48 h and then ground to a fine power using a mortar bowl and pestle. Each sample was acidified using 1 N of hydrochloric acid (HCl) and left overnight or until the reaction was complete. Samples were then centrifuged at approximately 2500 rpm for four minutes and 1 N HCl was added until the sample was no longer reactive. Samples were then centrifuged again. Once the HCl was decanted, DI water was added, and the sample was spun using a vortex mixer before placing it back into the centrifuge. We repeated this rinse two more times prior to drying at 60 °C for 48 h. Samples were then homogenized and stored in glass scintillation vials until analyzed similar to Nifong^49^ at the UF Light Stable Isotope Mass Spec Lab by Dr. Jason Curtis (see Supplement for details).

### PCB analysis

Total PCB concentrations were quantified in surficial sediment cores (n = 3 mussel enclosures; n = 3 no-mussel enclosures), pseudofeces cores (n = 3), fiddler crabs (n = 3 mussel enclosures; n = 3 no-mussel enclosures), and marsh crabs (n = 3 mussel enclosures; n = 3 no-mussel enclosures) to determine the presence and concentration of PCBs. Due to analytical cost constraints, we did not measure mussel, cordgrass or diatom tissue for PCBs nor crabs from ambient plots. Instead, we prioritized sediment, pseudofeces and crab tissue samples given our primary interest in evaluating whether mussels influence sediment PCB concentrations and if this in turn drives PCB bioaccumulation in two common primary consumers in the system. Further, to ensure enough samples would be available for both PCB and stable isotope analyses, we analyzed samples from 3 of the 6 enclosures per treatment (mussels vs. no mussels), resulting in a total of 21 samples. For each crab sample, ~ 6 marsh crabs and ~ 15 fiddler crabs per enclosures (all whole crabs) were first chopped into smaller pieces using a knife and cutting board and then homogenized in Teflon™ containers using a ProScientific Tissue Homogenizer with a stainless-steel homogenization probe (PRO Homogenizer: PRO250). Sediment and pseudofeces samples (3 sediment cores and 3 pseudofeces cores per enclosure) were homogenized using a stainless-steel spatula. All homogenization tools were cleaned with Liquinox soap and rinsed with DI water, acetone, and dichloromethane (DCM) between samples to prevent cross-contamination.

We analyzed samples for a suite of 100 PCBs (Supplementary Table S1) for comparability with other studies in the area^24^. The suite include congeners associated with Aroclor 1268^7,24,38,50,51^. Further this suite of PCBs also included 12 dioxin-like coplanar PCBs [77, 81, 105, 114, 118, 123 (co-eluted with 107), 126, 156, 157, 167, 169, 189]. PCBs were analyzed according to Wirth et al.^24^, see Supplement for details. Data quality was assessed by using blanks, reagent and matrix spikes, and a Standard Reference Material (SRM; NIST 1944 New York/New Jersey Waterway Sediment). Eighty-four percent of all spiked analytes fell within our accepted range of recovery (100% ± 20%). Average recoveries of matrix spikes (sediment) were 109% (SD 19%) and reagent spikes (water) 110.9% (SD 14.7%). Thirty-three of the 100 PCB congeners analyzed were certified in the SRM and 91% of those congeners fell within 80–120% recovery.

### Statistical analyses

We analyzed the effects of experimental treatment on response variables (including diatom biomass, soil temperature, and porewater salinity) measured at multiple time points using repeated measures analysis of variance with date set as a random factor in the analysis (RM-ANOVA; Stata SE v 13.1). Within enclosures, to assess the separate and combined effects of each of the invertebrate functional groups (mussels, fiddler crabs, and marsh crabs) on cordgrass stem height and biomass, we ran a three-way ANOVA. All post hoc analyses were completed using Bonferroni-corrected Tukey’s HSD tests. Since the marsh crab grazing data was not normally distributed, we applied a Kruskall-Wallis test to determine if there were significant differences among treatments, after which a Dunn post-hoc test was used to determine which treatments were significantly different.

We analyzed the difference among δ^15^N and among δ^13^C within each species across treatments using Kruskal–Wallis, a non-parametric method since neither response variable was normally distributed. We then used a t-test to evaluate whether δ^15^N and δ^13^C values differ by crab identity (fiddler crab and marsh crab).

To analyze the effect of experimental treatment on PCB_T_ concentrations, we ran a one-way ANOVA with treatment as the main factor. Post hoc analysis was completed using a Tukey’s HSD test. PCB homolog distribution was similarly evaluated using a one-way MANOVA with treatment as the main factor, followed by post-hoc pairwise comparisons to reveal variation among PCB homologs (Cl_4_–Cl_10_) in sediment and crabs from mussel and no mussel enclosures. TEQ values were also analyzed using a one-way ANOVA with treatment as the main factor and a Tukey’s HSD test for post hoc analysis. All findings are based on the reasonable assumption that PCB_T_ concentrations were similar across plots at the start of the experiment given the low variability in PCB_T_ concentrations and homolog compositions observed in the well-distributed plots without mussels (Fig. 1b).

## Supplementary Information

Supplementary Information

## Data Availability

Data associated will be freely available through the Georgia Coastal Ecosystems LTER Data Portal (https://gce-lter.marsci.uga.edu/portal/).

## References

[1] National Oceanic and Atmospheric Administration. What are PCBs? https://oceanservice.noaa.gov/facts/pcbs.html.

[2] United States Environmental Protection Agency. *EPA’s Final PCB Ban Rule: Over 100 Questions & Answers To Help You Meet These Requirements *(1979).

[3] Kim KH, Kabir E, Jahan SA (2017). Exposure to pesticides and the associated human health effects. Sci. Total Environ..

[4] Kaiser J, Enserink M (2000). Treaty takes a POP at the dirty dozen. Science.

[5] Jamieson AJ, Malkocs T, Piertney SB, Fujii T, Zhang Z (2017). Bioaccumulation of persistent organic pollutants in the deepest ocean fauna. Nat. Ecol. Evol..

[6] Desforges JP (2018). Predicting global killer whale population collapse from PCB pollution. Science.

[7] Pulster EL, Smalling KL, Zolman E, Schwacke L, Maruya KA (2009). Persistent organochlorine pollutants and toxaphene congener profiles in bottlenose dolphins (Tursiops truncatus) frequenting the Turtle/Brunswick River Estuary (TBRE) in coastal Georgia USA. Environ. Toxicol. Chem..

[8] Borgå K, Fisk AT, Hoekstra PF, Muir DCG (2004). Biological and chemical factors of importance in the bioaccumulation and trophic transfer of persistent organochlorine contaminants in arctic marine food webs. Environ. Toxicol. Chem..

[9] Hites RA (2004). Global Assessment of Organic Contaminants in Farmed Salmon. Science.

[10] Domingo JL, Bocio A (2007). Levels of PCDD/PCDFs and PCBs in edible marine species and human intake: a literature review. Environ. Int..

[11] Prince KD, Taylor SD, Angelini C (2020). A global, cross-system meta-analysis of polychlorinated biphenyl biomagnification. Environ. Sci. Technol..

[12] Knezovich JP, Harrison FL, Wilhelm RG (1987). The bioavailability of sediment-sorbed organic chemicals: a review. Water. Air. Soil Pollut..

[13] Björk M (1995). Bioavailability and uptake of hydrophobic organic contaminants in bivalve filter-feeders. Ann. Zool. Fennici.

[14] Kannan K (1998). Bioaccumulation and toxic potential of extremely hydrophobic polychlorinated biphenyl congeners in biota collected at a superfund site contaminated with Aroclor 1268. Environ. Sci. Technol..

[15] Viarengo A, Canesi L (1991). Mussels as biological indicators of pollution. Aquaculture.

[16] Granberg ME, Gunnarsson JS, Hedman JE, Rosenberg R, Jonsson P (2008). Bioturbation-driven release of organic contaminants from baltic sea sediments mediated by the invading polychaete marenzelleria neglecta. Environ. Sci. Technol..

[17] Bosworth WS, Thibodeaux LJ (1990). Bioturbation: A facilitator of contaminant transport in bed sediment. Environ. Prog..

[18] Björk M, Gilek M, Kautsky N, Näf C (2000). In situ determination of PCB biodeposition by Mytilus edulis in a Baltic coastal ecosystem. Mar. Ecol. Prog. Ser..

[19] Corsolini S, Sarà G (2017). The trophic transfer of persistent pollutants (HCB, DDTs, PCBs) within polar marine food webs. Chemosphere.

[20] Perga ME, Nellier Y-M, Cottin N, Fanget P, Naffrechoux E (2017). Bioconcentration may be favoured over biomagnification for fish PCB contamination in high altitude lakes. Inl. Waters.

[21] Smith JM, Frey RW (1985). Biodeposition by the ribbed mussel Geukensia demissa in a salt marsh, Sapelo Island Georgia. J. Sediment. Res..

[22] Crotty SM (2018). Foundation species patch configuration mediates salt marsh biodiversity, stability and multifunctionality. Ecol. Lett..

[23] Jones, C. G., Lawton, J. H. & Shachak, M. Organisms as ecosystem engineers. In *Ecosystem Management* 130–147 (Springer, 1994). 10.1007/978-1-4612-4018-1_14.

[24] Wirth EF (2014). Distribution and sources of PCBs (Aroclor 1268) in the Sapelo Island National Estuarine Research Reserve. Environ. Monit. Assess..

[25] Robinson GL, Mills GL, Lindell AH, Schweitzer SH, Hernandez SM (2015). Exposure to mercury and Aroclor 1268 congeners in least terns (Sternula antillarum) in coastal Georgia, USA. Environ. Sci. Process. Impacts.

[26] Backer L (2019). Environmental contaminants in coastal populations: Comparisons with the National Health and Nutrition Examination Survey (NHANES) and resident dolphins. Sci. Total Environ..

[27] Angelini C, van Montfrans SG, Hensel MJS, He Q, Silliman BR (2018). The importance of an underestimated grazer under climate change: how crab density, consumer competition, and physical stress affect salt marsh resilience. Oecologia.

[28] Vu HD, Pennings SC (2018). Predators mediate above- vs belowground herbivory in a salt marsh crab. Ecosphere.

[29] Angelini C (2015). Foundation species’ overlap enhances biodiversity and multifunctionality from the patch to landscape scale in southeastern United States salt marshes. Proc. R. Soc. B Biol. Sci..

[30] Peterson BJ, Howarth RW (1986). Sulfur, carbon, and nitrogen isotopes used to trace organic matter flow in the salt-marsh estuaries of Sapelo Island Georgia. Ecology.

[31] MacKenzie RA, Cormier N, Demopoulos AW (2020). Estimating the value of mangrove leaf litter in sesarmid crab diets: the importance of fractionation factors. Bull. Mar. Sci..

[32] Sherr EB (1982). Carbon isotope composition of organic seston and sediments in a Georgia salt marsh estuary. Geochim. Cosmochim. Acta..

[33] Post DM (2002). Using stable isotopes to estimate trophic position: models, methods, and assumptions. Ecology.

[34] Froese, R. & Pauly, D. Fishbase. www.fishbase.org (2019).

[35] Tomkins IR (1965). The Willets of Georgia and South Carolina. Wilson Bull..

[36] Peters KM, McMichael RH (1987). Early life history of the red drum, Sciaenops ocellatus (Pisces: Sciaenidae), in Tampa Bay Florida. Estuaries.

[37] Beyer, A. & Marek, B. Environmental fate and global distribution of polychlorinated biphenyls. *Rev. Environ. Contam. Toxicol.* **201**, (2009). 10.1007/978-1-4419-0032-6_5 19484591

[38] Maruya K, Lee R (1998). Aroclor 1268 and toxaphene in fish from a Southeastern US Estuary. Environ. Sci. Technol..

[39] Mrozek E, Seneca ED, Hobbs LL (1982). Polychlorinated biphenyl uptake and translation by Spartina alterniflora loisel. Water. Air. Soil Pollut..

[40] Long ER, Macdonald DD, Smith SL, Calder FD (1995). Incidence of adverse biological effects within ranges of chemical concentrations in marine and estuarine sediments. Environ. Manage..

[41] Van den Berg M (2006). The 2005 World Health Organization reevaluation of human and mammalian toxic equivalency factors for dioxins and dioxin-like compounds. Toxicol. Sci..

[42] United States Environmental Protection Agency. *Toxics Release Inventory Dioxin and Dioxin-like Compounds Toxic Equivalency ( TEQ ) Data Files Format Documentation v15*. https://www.epa.gov/sites/production/files/2016-08/documents/tri_teq_data_files_format_v15_final.pdf (2016).

[43] Karouna-Renier NK, Snyder RA, Allison JG, Wagner MG, Ranga Rao K (2007). Accumulation of organic and inorganic contaminants in shellfish collected in estuarine waters near Pensacola, Florida: contamination profiles and risks to human consumers. Environ. Pollut..

[44] Clark PF (2009). Dioxin and PCB contamination in Chinese mitten crabs: human consumption as a control mechanism for an invasive species. Environ. Sci. Technol..

[45] Lu Q (2015). The distribution of polychlorinated biphenyls (PCBs) in the River Thames Catchment under the scenarios of climate change. Sci. Total Environ..

[46] Crotty S, Angelini C (2020). Geomorphology and species interactions hierarchically structure the self-organization and landscape effects of a salt marsh facilitation cascade.

[47] Angelini C (2016). A keystone mutualism underpins resilience of a coastal ecosystem to drought. Nat. Commun..

[48] Bertness MD, Grosholz E (1985). Population dynamics of the ribbed mussel. Geukensia demissa : the costs and benefits of an aggregated distribution..

[49] Nifong JC (2016). Trophic ecology of American Alligator in Estuary. Bull. Florida Museum Nat. Hist..

[50] Balmer BC (2011). Relationship between persistent organic pollutants (POPs) and ranging patterns in common bottlenose dolphins (Tursiops truncatus) from coastal Georgia, USA. Sci. Total Environ..

[51] Kucklick J (2011). Bottlenose dolphins as indicators of persistent organic pollutants in the western North Atlantic Ocean and northern Gulf of Mexico. Environ. Sci. Technol..

