# Supplementary material for: Mussels drive polychlorinated biphenyl (PCB) biomagnification in a coastal food web
Source: Sci Rep. 2021 Apr 28;11:9180. doi: 10.1038/s41598-021-88684-9 (PMC8080837; doi:10.1038/s41598-021-88684-9)
Supplement: Supplementary file 1 — Supplementary Information [file 41598_2021_88684_MOESM1_ESM.pdf]

Supplementary Materials for

Mussels drive polychlorinated biphenyl (PCB) biomagnification in a coastal food web

Kimberly D. Prince*, Sinead M. Crotty, Alexa Cetta, Joseph J. Delfino, Todd M. Palmer, Nancy D. Denslow, Christine Angelini

Correspondence to: Kprince@ufl.edu

This PDF file includes:

Supplementary Methods
Supplementary Figure S1 and S2
Supplementary Tables S1 to S3

Supplementary Methods

Study site

We conducted the field experiment in a cordgrass dominated saltmarsh platform located on Blythe Island, Georgia (31°10′47.8″N, 81°32′02.1″W; Supplementary Fig. S1B). This site experiences diurnal tides and is inundated for 0-5 hours per tide. Porewater salinity ranged from 20‰ to 47‰ (35 ± 2 ‰) and average air temperatures ranged from 21°C in April to an average of 27°C in August 2017. Blythe Island is adjacent to Turtle River, where chemical contaminant data has been well documented[1–3]. PCBs in the area are generally attributed to Aroclor 1268. Aroclor 1268 is a highly chlorinated mixture of PCBs that was previously used by LCP from 1955 – 1994 to lubricate high voltage equipment during the manufacturing of chloralkali. After 39 years of discharging hazardous waste into holding pits adjacent to the saltmarsh and directly into Purvis Creek, a tributary of the Turtle River, the U.S. EPA designated the LCP facility and 813 acres of impacted tidal marshlands and uplands a Superfund site in 1996[2].

Remediation via burying and dredging sediment was conducted at the Superfund site between 1994 to 1999 on 136 of the 813 identified impacted acres[4]. Despite these efforts, PCBs continue to be documented in the sediment[2] as well as in benthic[5] and pelagic[3,6–8] biota throughout the Turtle-Brunswick River estuary and adjacent barrier islands[3,9] that are intensively commercially and recreationally fished despite fishing advisories[3,10]. Due to the available research in the area, Blythe Island served as an ideal location to assess how common benthic salt marsh macro-invertebrates, and suspension-feeding bivalves in particular, may influence PCB assimilation into coastal food webs.

Stable isotope sample preparation

Similar to Nifong (2016)[11], stable isotope values ($\delta^{13}C$ and $\delta^{15}N$) are expressed in standard per mil notation (‰):

$$\delta X\,(‰) = [R_{sample} / R_{standard} - 1] \times 1000$$

where X is the element of interest and R is the ratio of heavy to light isotopes ($^{13}C/^{12}C$ or $^{15}N/^{14}N$) of the sample and standard (Vienna Pee Dee Belemnite used for $\delta^{13}C$ and Atmospheric Nitrogen-AIR for $\delta^{15}N$). Instrument accuracy was measured and corrected for each sample run using five measures of in-lab standard USGS- 40 (l-glutamic acid) with $\delta^{13}C$ = -26.39 and $\delta^{15}N$ = -4.52. Analytical instrument error for USGS-40 was 0.08 for $\delta^{15}N$ and 0.10 for $\delta^{13}C$ across all runs.

PCB sample extraction

Five grams of each homogenized fiddler and marsh crab sample from their respective mussel and no mussel enclosures were weighed into ~30 g of anhydrous sodium sulfate and 10g of each homogenized sediment and pseudofeces sample was weighed into ~27.2 g of anhydrous sodium sulfate. All samples were ground until dry with a mortar and pestle. Samples were then transferred into 22 mL accelerated solvent extraction (ASE) cells, spiked with a suite of labeled ^{13}C-PCB congeners (Wellington Laboratories) and extracted using pressurized fluid extraction (ASE 200, Dionex Inc.) with 100 % DCM for tissue samples and 1:1(v/v) acetone:DCM for sediment and pseudofeces. The resulting extract was filtered through additional sodium sulfate for residual water removal. The sample was then reduced under nitrogen (TurboVap®) and underwent size exclusion chromatography through an SX-3 gel permeation chromatography column (GPC, J2 Scientific, Inc.) to remove lipids and pigments. Post-GPC extracts were solvent exchanged under nitrogen (TurboVap®) from DCM to hexane (final volume of 0.5mL). Lastly, we used alumina solid phase extraction (SPE) (~1.1 – 1.15g of 5% water-deactivated alumina) as a final cleanup step, resulting in a final volume of ~0.5mL. A recovery standard (δ-hexachlorocyclohexane) was then added prior to instrumental analysis to evaluate internal standard recoveries.

Instrumental analysis and data quality

Samples were analyzed for 100 PCB congeners (Supplementary Table S1) using an Agilent 6890 gas chromatograph (GC) equipped with a 5973 Mass Selective Detector operated using electron impact ionization. Data was acquired in selected ion monitoring mode. The GC included a DB-XLB column (J&W; 30 m × 0.25 mm diameter × 0.25 μm) and a programmable temperature vaporization inlet. Congener concentrations above our analyte specific method detection limit[12] were summed for a total PCB_T concentration in each sample.

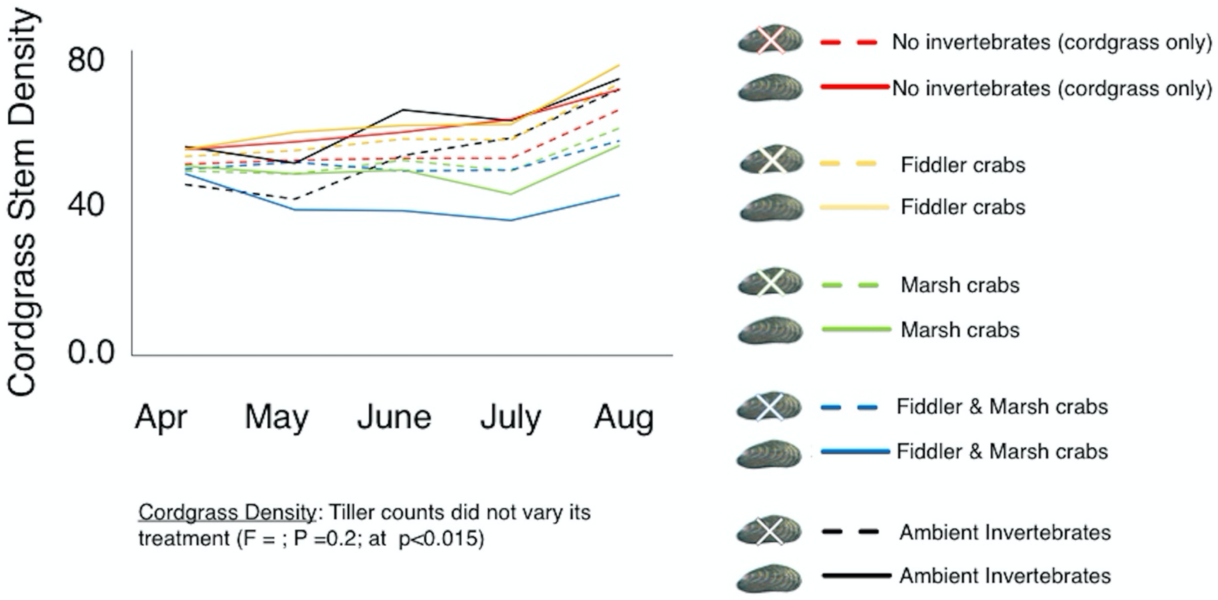

Supplementary Figure S1: Cordgrass density. Stem density was recorded prior to the experimental setup and every 4-6 weeks over the 5-month experiment. The data are reported as the mean ± standard error of 6 replicate plots per date.

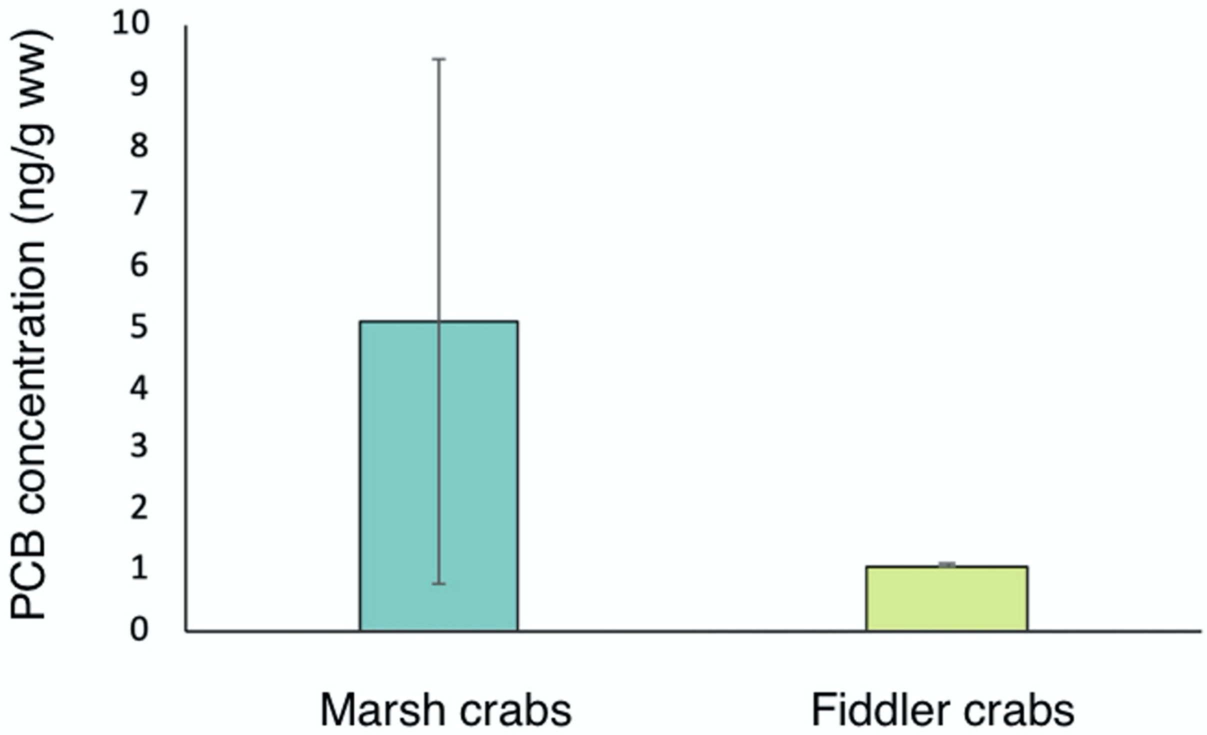

Supplementary Figure S2: Baseline PCB concentrations for crabs sampled from Sapelo Island, GA. PCBs (ng/g ww) are lipid normalized and shown as mean ± standard error of 3 extractions taken from a homogenized composite of whole marsh crabs (n=143) and a homogenized composite of whole fiddler crabs (n=143). The large standard error for marsh crabs is due to the fact that that composite of crabs were sampled from both on and off mussel mounds.

Supplementary Table S1. PCB congeners sought in various matrices during this study. For each sample, those detected above the analyte specific method detection limit were summed and reported as a total PCB concentration for that sample. Co-planars analyzed are in bold and congeners that represent PCB_{1268} are italicized.

PCB Congeners		
1	**81**	159
2	82	163
3	84/89/92	164
5	87	165
8	88/95	**167**
9	90/101	**169**
12	99	170
15	103	172
18	104	*174*
20	**105**	177
26	106	*180*
28	107/**123**	*183*
29	108	184
31	110	*187*
37	**114**	188
44	115	**189**
45	**118**	190
47	119	193

48	**126**	*194*
49	128	195
50	130	*196*/203
52	132/153/168	198
56	138	*200* / IUPAC 201
60	141	*201* / IUPAC 199
61	146	*202*
63/76	149	*206*
66	151	*207*
69	154	*208*
70	**156**	*209*
74	**157**	
**77**	158	

Supplementary Table S2. Effects of mussels on dioxin-like toxicity. Experimental treatment effects on the concentration of five coplanar PCBs pg/g ww (dw in parentheses for sediment and pseudofeces). ND = not detected above method detection limit. If a coplanar was detected in multiple treatment enclosures concentrations are shown as mean ± standard error. TEF values per coplanar PCB is provided in the first row. The average total coplanar concentration per treatment and their toxic equivalency values from coplanar PCBs in sediment, fiddler crabs and marsh crabs are also shown as the mean of 3 replicate enclosures ± standard error. The last column contains TEQ values reported as mean; min – max pg/g ww (dw in parentheses for sediment and pseudofeces) for dioxin-like PCBs from this study as well as from other invertebrates previously reported on in the literature.

Citation	Location	Sample	PCB 105 TEF: 0.00003	PCB 118 TEF: 0.00003	PCB 126 TEF: 0.1	PCB 169 TEF: 0.03	PCB 189 TEF: 0.00003	Total PCB_{DL}	TEQ
This study	Blythe Island, GA	Sediment (no mussels)	ND	ND	ND	235.0±76.6 (828.1± 214.8)	ND	235.0±76.6 (828.1± 241.8)	7.0; 0.01 – 75.1 (24.8; 12.1 – 33.1)
This study	Blythe Island, GA	Sediment (mussels)	120.8 (455.6)	261.1±43.4 (7,467.9 ± 1,513.5)	ND	1,908.1 ± 473.7 (994.6 ± 1,36.8)	610.6 (2,302.3)	1,777.0± 860.8 (1,3263.4 ± 9,332.0)	38.2; 3.1 – 11.1 (149.4;0.04 – 269.5)
This study	Blythe Island, GA	Pseudofeces (mussels)	ND	111.7 (403.6)	ND	741.8± 65.1 (2,691.9 ± 241.2)	ND	779.1±69.6 (2,826.4 ± 254.1)	22.3; 19.2 – 25.9 (80.8; 69.7 – 94.4)
This study	Blythe Island, GA	Marsh crabs (no mussels)	ND	180.1	ND	465.2±163.0	ND	525.3±194.6	14.0; 4.2 – 19.8
This study	Blythe Island, GA	Marsh crabs (mussels)	ND	479.6±131.9	ND	4,396.9 ±195.1	ND	4,716.7± 236.8	131.9; 120.3 – 138.9
This study	Sapelo Island, GA	Marsh crab (baseline)	ND	ND	ND	34.2	ND	34.2	1.0
This study	Blythe Island, GA	Fiddler crabs (no mussels)	ND	109.5±3.0	ND	864.1±194.8	19.0	943.4±233.5	25.9; 14.9 - 28
This study	Blythe Island, GA	Fiddler crabs (mussels)	ND	ND	266.0	738.5± 143.6	ND	827.2±76.22	22.2; 14.5 - 29.4
This study	Sapelo Island, GA	Fiddler crab (baseline)	ND	ND	ND	39.2	ND	39.2	1.2
Clark et al. 2009	Thames Estuary, UK	Chinese mitten crabs (*Eriocheir sinensis*; hepatopancreas/ gonad)	NA	NA	NA	NA	NA	NA	39.6; 13.1 – 111.1

Karouna-Renier et al. 2007	Pensacola, Florida	Blue crabs (*Callinectes sapidus* ; hepatopancreas)	NA	NA	NA	NA	NA	NA	9.9; 1.7 – 25.8
Karouna-Renier et al. 2007	Pensacola, Florida	Blue crabs (*Callinectes sapidus*; muscle)	NA	NA	NA	NA	NA	NA	0.23; 0.04 - 0.69
Okumura et al. (2004)	Sendai Bay (Japan)	Brown shrimp (*Crangon sp.*; whole body)	NA	NA	NA	NA	NA	NA	0.39; 0.35 - 0.43
Yitalo et al. 1999	Atlantic SE USA	American lobster (Homarus americanus; muscle)	NA	NA	NA	NA	NA	NA	0.25; 0.06 – 0.51
Yitalo et al. 1999	Pacific NW USA	Dungeness crab (*Cancer magister;* muscle)	NA	NA	NA	NA	NA	NA	0.35; 0.07 – 1.30

Supplementary Table S3. Summary of response variables measured in each treatment. A "Y" signifies that the response variable was measured for the specified treatment. Cordgrass and benthic algae variables, porewater salinity, and soil temperature were measured in every plot per treatment (n=6 replicate plots per treatment), invertebrate densities were measured in every enclosure with invertebrates, stable isotopes were measured in every plot of identified treatments with the exception of some plots used for PCB analyses due to lack of enough available material. For all PCB related response variables, half of the replicate enclosures per treatment (n=3) were used for these analyses (Supplementary Fig. S1C).

Response variable	No invertebrates	Fiddler crabs only	Marsh crabs only	Fiddler crabs and marsh crabs	Mussels only	Fiddler crabs and mussels	Marsh crabs and mussels	Fiddler crabs, marsh crabs, and mussels
Cordgrass stem height	Y	Y	Y	Y	Y	Y	Y	Y
Cordgrass biomass	Y	Y	Y	Y	Y	Y	Y	Y
Live and dead stem biomass	Y	Y	Y	Y	Y	Y	Y	Y
Crab density (burrows)		Y	Y	Y		Y	Y	Y
Mussel density (live/dead)					Y	Y	Y	Y
Soil temperature	Y	Y	Y	Y	Y	Y	Y	Y
Porewater salinity	Y	Y	Y	Y	Y	Y	Y	Y
Diatom biomass	Y	Y	Y	Y	Y	Y	Y	Y
Stable isotopes		Y	Y	Y		Y	Y	Y
Total PCBs	Y	Y	Y		Y	Y	Y	
PCB1268	Y	Y	Y		Y	Y	Y	
PCB homologs	Y	Y	Y		Y	Y	Y	
Dioxin-like coplanar PCBs	Y	Y	Y		Y	Y	Y	
TEQs	Y	Y	Y		Y	Y	Y	

1. Maruya, K. A. & Lee, R. F. Biota-sediment accumulation and trophic transfer factors for extremely hydrophobic polychlorinated biphenyls. *Environ. Toxicol. Chem.* **17**, 2463–2469 (1998).
2. Kannan, K., Maruya, K. A. & Tanabe, S. Distribution and characterization of polychlorinated biphenyl congeners in soil and sediments from a superfund site contaminated with Aroclor 1268. *Environ. Sci. Technol.* **31**, 1483–1488 (1997).
3. Wirth, E. F. *et al.* Distribution and sources of PCBs (Aroclor 1268) in the Sapelo Island National Estuarine Research Reserve. *Environ. Monit. Assess.* **186**, 8717–8726 (2014).
4. United States Environmental Protection Agency. LCP Chemicals Georgia Brunswick, GA. https://cumulis.epa.gov/supercpad/CurSites/csitinfo.cfm?id=0401634&msspp=med.
5. Kannan, K. *et al.* Bioaccumulation and toxic potential of extremely hydrophobic polychlorinated biphenyl congeners in biota collected at a superfund site contaminated with Aroclor 1268. *Environ. Sci. Technol.* **32**, 1214–1221 (1998).
6. Balmer, B. C. *et al.* Relationship between persistent organic pollutants (POPs) and ranging patterns in common bottlenose dolphins (Tursiops truncatus) from coastal Georgia, USA. *Sci. Total Environ.* **409**, 2094–101 (2011).
7. Pulster, E. L., Smalling, K. L., Zolman, E., Schwacke, L. & Maruya, K. a. Persistent organochlorine pollutants and toxaphene congener profiles in bottlenose dolphins (Tursiops truncatus) frequenting the Turtle/Brunswick River Estuary (TBRE) in coastal Georgia, USA. *Environ. Toxicol. Chem.* **28**, 1390–1399 (2009).
8. Kucklick, J. *et al.* Bottlenose dolphins as indicators of persistent organic pollutants in the western North Atlantic Ocean and northern Gulf of Mexico. *Environ. Sci. Technol.* **45**, 4270–4277 (2011).
9. Balthis, L. *et al.* Support for Integrated Ecosystem Assessments of NOAA's National Estuarine Research Reserve System (NERRS): Assessment of Ecological Condition and Stressor Impacts in Subtidal Waters of the Sapelo Island National Estuarine Research Reserve. *NOAA Tech. Memo. NOS NCCOS* **150**, 79 (2012).
10. Schalles, J. F., Hladik, C. M., Lynes, A. A. & Pennings, S. C. Landscape Estimates of Habitat Types, Plant Biomass, and Invertebrate Densities in a Georgia Salt Marsh. *Oceanography* **26**, 88–97 (2013).
11. Nifong, J. C., Layman, C. A. & Silliman, B. R. Size, sex and individual-level behaviour drive intrapopulation variation in cross-ecosystem foraging of a top-predator. *J. Anim. Ecol.* **84**, 35–48 (2015).
12. Ragland, J. M., Liebert, D. & Wirth, E. Using Procedural Blanks to Generate Analyte-Specific Limits of Detection for Persistent Organic Pollutants Based on GC-MS Analysis. *Anal. Chem.* **86**, 7696–7704 (2014).
